# High post-exposure prophylaxis uptake but low completion rates and HIV testing follow-up in health workers, Harare, Zimbabwe

**DOI:** 10.3855/jidc.12214

**Published:** 2021-04-30

**Authors:** Fadzai Mushambi, Collins Timire, Anthony D Harries, Hannock Tweya, Tafadzwa Priscilla Goverwa-Sibanda, Stanley Mungofa, Tsitsi Apollo

**Affiliations:** 1 Emergency Department, Parirenyatwa Group of Hospitals, Harare, Zimbabwe; 2 Ministry of Health and Child Care, National AIDS and TB Program, Harare, Zimbabwe; 3 International Union Against Tuberculosis and Lung Disease (The Union), Zimbabwe; 4 The Union, Paris, France; 5 London School of Hygiene and Tropical Medicine, London, United Kingdom; 6 The Lighthouse Trust, Lilongwe, Malawi; 7 AIDS Healthcare Foundation, Harare, Zimbabwe; 8 Ministry of Health and Child Care, Mpilo Hospital, Bulawayo, Zimbabwe; 9 Health Services Board, Harare, Zimbabwe

**Keywords:** Post exposure prophylaxis, health care workers, Zimbabwe, occupational injury, operational research, SORT IT

## Abstract

**Introduction:**

Health care workers (HCWs), especially from sub-Saharan Africa, are at risk of occupational exposure to HIV. Post exposure prophylaxis (PEP) can reduce this risk. There is no published information from Zimbabwe, a high HIV burden country, about how PEP works. We therefore assessed how the PEP programme performed at the Parirenyatwa Hospital, Harare, Zimbabwe, from 2017–2018.

**Methodology:**

This was a cohort study using secondary data from the staff clinic paper-based register. The chi square test and relative risks were used to assess associations.

**Results:**

There were 154 HCWs who experienced occupational injuries. The commonest group was medical doctors (36%) and needle sticks were the most frequent type of occupational injury (74%). The exposure source was identified in 114(74%) occupational injuries: 91% of source patients were HIV-tested and 77% were HIV-positive. All but two HCWs were HIV-tested, 148 were eligible for PEP and 142 (96%) started triple therapy, all within 48 hours of exposure. Of those starting PEP, 15 (11%) completed 28 days, 13 (9%) completed < 28 days and in the remainder PEP duration was not recorded. There were no HCW characteristics associated with not completing PEP. Of those starting PEP, 9 (6%) were HIV-tested at 6-weeks, 3 (2%) were HIV-tested at 3-months and 1 (< 1%) was HIV-tested at 6-months: all HIV-tests were negative.

**Conclusions:**

While uptake of PEP was timely and high, the majority of HCWs failed to complete the 28-day treatment course and even fewer attended for follow-up HIV-tests. Various changes are recommended to promote awareness of PEP and improve adherence to guidelines.

## Introduction

Post exposure prophylaxis (PEP) against human immunodeficiency virus (HIV) infection dates back to the early 1990s when only limited antiretroviral treatment (ART) was available to treat people with HIV. A case-control study in 1997 showed that health care workers (HCWs) who took zidovudine monotherapy after needle stick exposure were 80% less likely to become HIV-infected [1]. Since then, observational evidence has accumulated about the risks of HIV transmission in relation to the type of occupational injury (percutaneous, mucous membrane), the characteristics of the source patient (HIV-positive, severely immunosuppressed), the timing and duration of PEP and the types of regimen used [2].

This evidence has informed World Health Organization (WHO) Guidelines on PEP over the last few years [3–5]. Key recommendations include the need to offer and initiate PEP as soon as possible, and preferably within 72 hours, in all individuals who have an exposure that has the potential for HIV transmission. For adults and adolescents, a three drug regimen for 28 days is preferred consisting of tenofovir (TDF) and lamivudine (3TC) as the backbone nucleos(t)ide regimen combined with lopinavir / ritonavir (LPV/r) or atazanavir / ritonavir (ATV/r). Follow-up care is context specific, and in a developed country would consist of a 2-week review to check on medication toxicity and adherence and repeat HIV screening after completion of the 28-day course at one month, four months and six-months [6].

Published studies from African countries in the last ten years point to a lack of awareness and uptake of PEP amongst HCWs and students after occupational injury to HIV [7–13]. This is further compounded by poor follow-up and lack of HIV testing after completion of medication. There has been one published study on PEP from Zimbabwe but this was not in HCWs and assessed PEP in sexual assault victims in a general hospital [14]. This study found sub-optimal administration of PEP with only 51% of sexual assault victims receiving appropriate medication. Given that Zimbabwe is a high HIV burden country, more needs to be learnt about PEP amongst its HCWs.

The Parirenyatwa Group of Hospitals includes the University Teaching Hospital in Harare which runs a PEP programme for its health-care and non-health care staff with a paper-based register to track what happens after occupational injury. There is anecdotal evidence that more than half of the HCWs who start PEP do not complete the treatment course nor attend scheduled staff clinic visits for follow-up HIV testing. The extent of and characteristics associated with this attrition from treatment and follow-up are unclear. We therefore undertook a formal study to assess how the PEP programme performed at the Parirenyatwa Group of Hospitals, Harare, Zimbabwe, between January 2017 and December 2018. In HCWs who received an occupational injury and presented for PEP at the staff clinic, the objectives were to determine: i) their characteristics and occupational injuries, ii) hospital management of PEP in terms of establishing the HIV status of the exposure source, HIV testing of HCWs, the initiation and timing of PEP, and follow-up in terms of completing treatment and repeat HIV testing and iii) factors associated with not completing a full course of PEP treatment.

## Methodology

### Study design

This was a cohort study using secondary data.

### Setting

#### General Setting

Zimbabwe is a land-locked country in southern Africa with a population of about 13 million [15]. It is a low-income country with a gross domestic product per capita of US$ 924 compared to US$ 1,588 for Sub-Saharan Africa as a whole [16]. The country has for several decades suffered from a generalized HIV epidemic. Current HIV-prevalence is estimated at 14.6% and annual HIV incidence at 0.45% among the 15–64 year old adult population according to the concluded Zimbabwe Population-based HIV Impact Assessment (ZIMPHIA) survey [17]. The country has made good progress in its response to the HIV epidemic. According to the ZIMPHIA survey, 74% of PLHIV in the country know their HIV status, 87% of those diagnosed with HIV receive ART and 87% of those on ART are virally suppressed [17].

#### Specific Setting

The University Teaching Hospital in Harare has approximately 1,000 beds and about 10,000 HCWs, including clinical staff, nurses, medical and nursing students, nurse aids, general hands and cleaners. There is a staff clinic which opens 8 hours daily during weekdays for all HCWs, and an opportunistic infections (OI) clinic which also serves the general population of PLHIV.

At the hospital, HCWs who experience an HIV occupational injury are assessed for PEP at the staff clinic in line with national infection prevention and control guidelines [18]. Occupational injury is defined as needle stick injury, splash to the mucous membranes, and cuts or bites that may result in HIV transmission. The HIV status of the exposure source is ascertained either through documented proof of being HIV positive or being newly tested for HIV using the Determine Rapid Test (Alere Determine HIV-1/2 Ag/Ab Combo). Whether that result is positive or negative, the HCWs are counselled about PEP and tested for HIV infection according to National HIV testing guidelines [19]. Those testing positive for HIV infection are referred for initiation of first-line ART. Those who test negative are offered and started on PEP which should be given within 72 hours of the occupational injury. PEP consists of triple therapy with TDF, 3TC and ATV/r, taken as two tablets daily for a total of 28 days [19]. Initially, a three day supply is given at the staff clinic, with the remaining 25 days of medication given all at once at the OI clinic.

At 6 weeks after starting PEP, a second HIV test is done. Any HCW testing HIV-positive at this stage is considered to have HIV infection that is not secondary to the occupational injury and he/she is initiated on ART. Those testing HIV-negative are retested at 3-months and again at 6-months after starting PEP to determine the effectiveness of the intervention. A positive HIV test at this stage is indicative of HIV infection secondary to occupational injury and these HCWs are eligible for compensation.

The process of administering, following up and monitoring HCWs on PEP is done by nurses in the staff clinic using a paper-based register.

### Study population

Health care workers who were registered after having an occupational injury at Parirenyatwa Hospital, Harare, Zimbabwe between January 2017 and December 2018 were included in the study.

### Data variables, source of data and data collection

Study data variables included: HCW registered for PEP; year; month; monthly sequence number; sex; age; job title; type of occupational injury; date and time of occupational injury; exposure source identified, HIV tested and HIV result; HCW HIV tested and HIV result; start of PEP; date and time of starting PEP; PEP regimen used; number of days of PEP (self-reported or as a result of telephone contact); HIV testing at 6 weeks, at 3 months and at 6 months with results. The data source was the PEP register in the staff clinic at the University Hospital. Data were extracted from the Register into an EpiData collection file between April and August 2019.

### Analysis and statistics

Data were entered and analysed using EpiData (version 3.1 for data entry and version 2.2.2.182 for data analysis, EpiData Association, Odense, Denmark). Frequencies and proportions were used to summarise categorical variables. Means and standard deviations were used to summarise continuous variables. Baseline characteristics of HCWs and types of occupational injury were compared with respect to not completing a full course of PEP using the chi square test (or Fisher’s exact test when cell frequency was < 5) and presented as relative risks (RR) and 95% confidence intervals (CI). Levels of significance were set at 5% (*p* < 0.05).

### Ethics

Permission for the study was obtained from the clinical director at Parirenyatwa Group of Hospitals. Ethics approval was obtained from the Medical Research Council of Zimbabwe (MRCZ/E/249) and the Ethics Advisory Group, International Union Against Tuberculosis and Lung Disease, Paris, France (EAG 45/19). As secondary data were used, the need for informed patient consent was waived.

## Results

There were 154 HCWs who experienced occupational injury, 87 in 2017 and 67 in 2018. Their mean age (SD) was 29.5 (6.3) years. HCW characteristics are shown in Table 1. Nearly two thirds were female, over 90% were aged 20–39 years and the commonest group experiencing occupational injury was medical doctors (36%). Needle sticks were the most frequent type of occupational injury in 74% of cases followed by splashes to the eyes in 19%.

**Figure 1.**
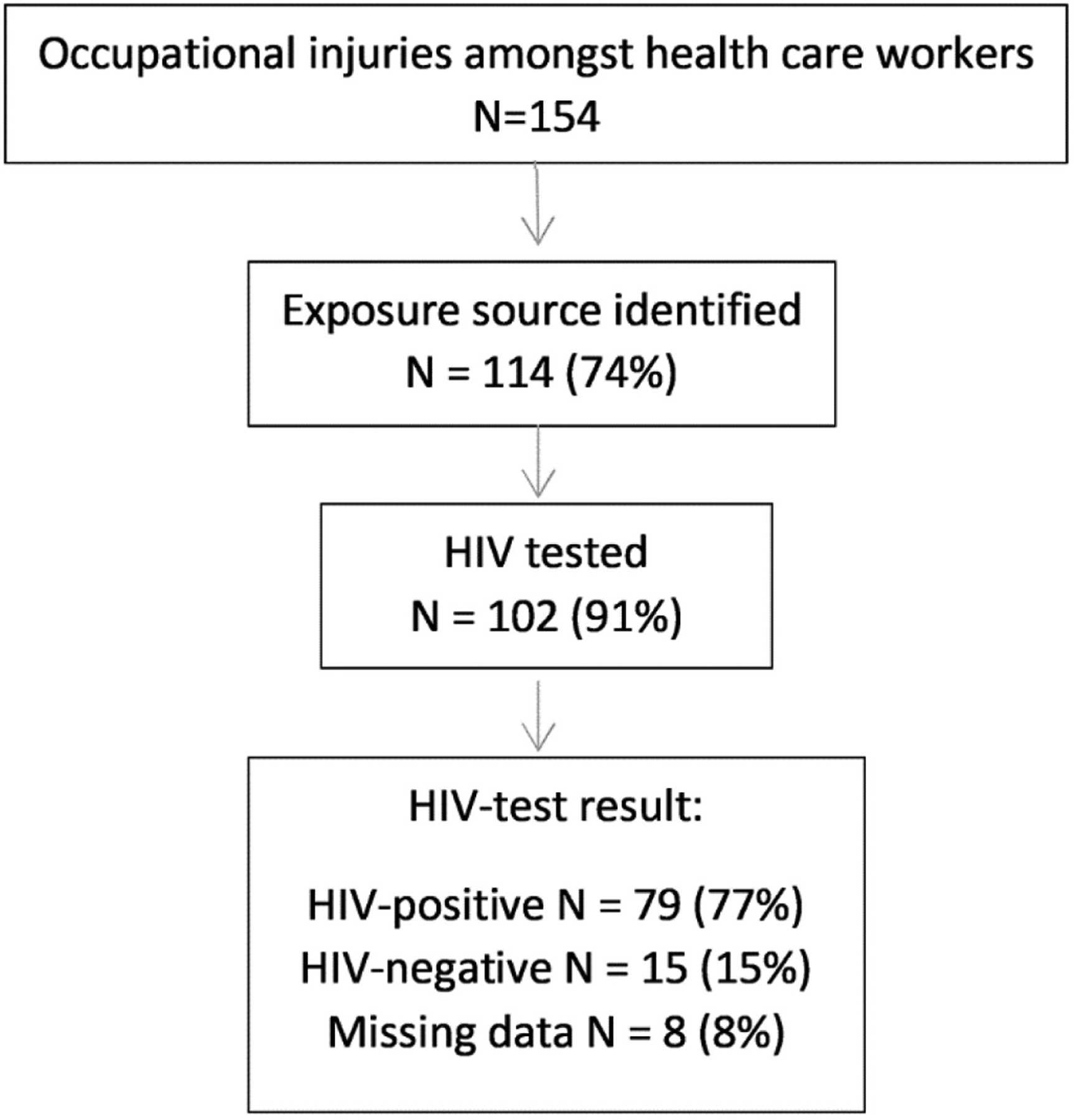
The exposure source responsible for the occupational injury: identification, HIV testing and HIV test results at Parirenyatwa Hospital, Harare, Zimbabwe: January 2017 to December 2018.

The source patient was identified in 114 (74%) occupational injuries. HIV testing and HIV test results for source patients are shown in Figure 1. Just over 90% of identified source patients were HIV tested, of whom 77% were HIV positive: there were missing data for HIV test results in 8 (8%) patients.

**Figure 2.**
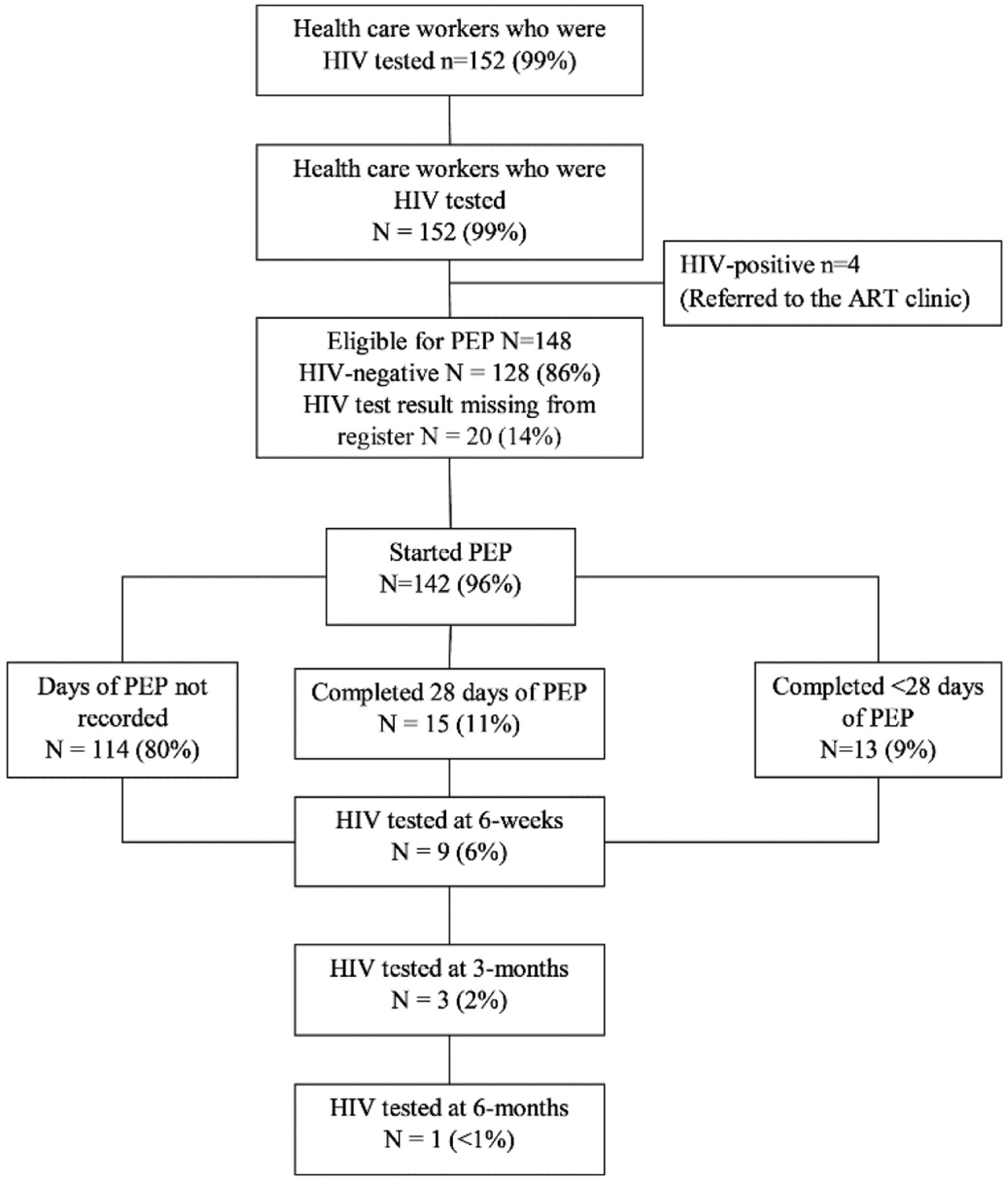
Management and follow-up of health care workers after occupational injury at Parirenyatwa Hospital, Harare, Zimbabwe: January 2017 to December 2018. ART = antiretroviral therapy; HIV = human immunodeficiency virus; PEP = post-exposure prophylaxis.

Management and follow-up of HCWs after occupational injury is shown in Figure 2. All except two HCWs were HIV tested. Of these, 4 (3%) were HIV positive. The remainder were eligible for PEP. Of those eligible for PEP, 86% were tested HIV negative and in the remainder the HIV test results were missing from the register. Of HCWs eligible for PEP, 142 (96%) started PEP: 110 (77%) started within 24 hours of occupational injury and 32 (23%) started between 24 and 48 hours of occupational injury. All HCWs took a regimen of TDF, 3TC and ATV/r as two tablets once a day. Of those who started PEP, 15 (11%) completed 28 days, 13 (9%) completed less than 28 days (median 3 days, range 1–14 days) and in the remainder the duration of PEP was not recorded. Of those who started PEP, 9 (6%) were HIV tested at 6 weeks, 3 (2%) were HIV tested at 3 months and 1 (< 1%) was HIV tested at 6 months: all HIV tests were negative.

Risk factors associated with not completing the 28-day course of treatment in those who started PEP are shown in Table 2. Altogether 127 (89%) HCWs that started PEP did not complete the course. There were no significant associations with failing to complete PEP treatment with respect to gender, age group, job title or type of occupational injury.

## Discussion

This is the first study in Zimbabwe to document the implementation of PEP amongst HCWs who experienced an occupational injury in a tertiary hospital in Harare. There were four main findings.

First, the main cadre of HCW affected was the medical doctor and the principal occupational injury was a needle stick. This pattern is similar to what has been described in other African health care facilities in Malawi, Tanzania and Nigeria [7,8,20]. In contrast, in Europe nurses appear to be more affected because of taking more responsibility for injections and venous cannulation [21]. Nearly three quarters of the exposure source patients were identified, with the majority HIV-tested. Of these, nearly 80% were HIV-positive pointing to the need for PEP in these circumstances. However, even if the source patient is HIV-negative, it is still recommended that the HCW be HIV tested and offered PEP because of the window period during which source persons may be highly infectious.

Second, the process of HIV testing and start of PEP for the HCWs worked well. All but two of the 154 HCWs were HIV tested, of whom four were HIV-positive. While most HCWs were HIV-negative, one in seven had no documented HIV result in the register. It is likely, however, that these HCWs were also HIV-negative as most of them started PEP treatment. The uptake of PEP was higher than that reported recently from Tanzania, Cameroon, Botswana and South Africa where uptake ranged from 12% to 78% [8,10,12,13,22,23]. All HCWs at the clinic started triple drug therapy within 48 hours in line with national guidelines. This is a faster uptake compared with South Africa [13], but similar to that reported from Malawi [7].

Third, the proportion of HCWs recorded as completing PEP was just over 10%, much lower than that recorded in other studies from Botswana, Tanzania and South Africa where rates of completion ranged from 23% to 71% [10,12,13,23,24]. The reasons are unclear as the register did not record why the PEP course was not completed, but it may have been due to stigma related to HCWs having to queue and collect medication at the OI clinic, failure to record PEP completion in the register, side effects of medication or belief that the 28-day course was unnecessary or not effective. Side effects of treatment were the most important reasons for discontinuing therapy in Botswana and South Africa [10,13].

Finally, follow-up HIV testing was poor with only one HCW having an HIV test at 6-months. This was worse than that observed in Tanzania and South Africa where follow-up HIV testing occurred in 54% and 28% of HCWs respectively [12,25]. The reasons for this poor follow-up are also unclear but may relate to stigma, workplace discrimination and fear of lack of confidentiality. In Botswana, HCWs preferred to be tested outside of their own health facility or the option of being able to HIV self-test [24]. However, service providers may have difficulties in knowing and recording the test results when individuals use this approach.

The strengths of the study were the large number of occupational injuries within a routine hospital setting over a two-year period. The conduct and reporting of the study also adhered to the Strengthening the Reporting of Observational Studies in Epidemiology (STROBE) [26]. However, there were some limitations that mainly related to poor recording of information in the register leaving it open to doubt as to whether HIV tests had not been done or had just not been recorded. We also do not know whether HCWs completing their 28-day course of PEP actually swallowed all their tablets. The implementation of the study in a tertiary health facility may also limit the generalisability of the findings to the country as a whole.

Despite these limitations, we can make some recommendations to improve the performance of the PEP programme. First, there are two changes that might help HCWs to complete PEP: i) instead of providing HCWs with an initial 3-day PEP supply, the staff clinic should make a full 28-day course of PEP available – this would simplify the process; ii) the HIV/AIDS programme should replace the protease inhibitor component, that is associated with many adverse events, with dolutegravir (DTG) which is much better tolerated – such a change has recently been recommended by WHO [5] and has already started to be implemented in Zimbabwe.

Second, mobile phone text message reminders could be sent regularly to HCWs about the schedule and the need to attend follow-up HIV tests. Short message service (SMS) texting is now a well-established intervention for improving clinic visits and medication adherence within HIV/AIDS programmes [27], and this practice is already finding its way into HCW’s lives in Zimbabwe [28]. There could be more flexibility about where HIV testing is carried out to allay concerns about privacy and confidentiality. The use of HIV self-testing, which has been found to be highly acceptable, feasible and accurate in sub-Saharan Africa [29], could also be explored.

Third, the completeness and accuracy of recording and reporting must improve and this could be facilitated by introducing data entry on a computer, backed up by a paper based register. Finally, much more attention needs to be paid to educating HCWs and health care students about occupational injuries and the benefits of PEP through formal introduction of the subject in student curricula, continuous medical education programmes, standardized video recordings and workshops [30].

## Conclusions

There were 154 HCWs who presented to the staff clinic at the University Teaching Hospital, Harare, Zimbabwe, with an occupational injury between January 2017 and December 2018. Most of those eligible for PEP started a standardized triple therapy treatment regimen, all within 48 hours of exposure. However, the proportion that completed the 28-day course of treatment was low at 11%. Even fewer HCWs attended for follow-up HIV tests, all of which were negative. Ways to improve the performance of the PEP programme and subsequent follow-up are discussed.

## Figures and Tables

**Table 1.** Characteristics of health care workers who experienced occupational injury at Parirenyatwa Hospital, Harare, Zimbabwe: January 2017 to December 2018.

Characteristics	n (%)
**Total**	154 (100)
**Gender**	
Male	59 (38)
Female	95 (62)
**Age group in years**	
< 20	3 (2)
20–29	76 (52)
30–39	57 (39)
40–49	7 (5)
≥ 50	2 (1)
Missing data	9 (6)
**Job title**	
Medical doctors	55 (36)
Medical students	14 (9)
Nurses	31 (20)
Student nurses and nurse aids	21 (14)
Dental and laboratory staff	5 (3)
Non clinical staff §	28 (18)
**Type of occupational injury / injury**	
Needle stick	113 (74)
Cuts from blades / glass	10 (7)
Splashes	29 (19)
Human bite	1 (<1)
Missing data	1 (<1)

§ includes cleaners, porters, messengers and others.

**Table 2.** Risk factors for not completing post exposure prophylaxis in health care workers who experienced occupational injury at Parirenyatwa Hospital, Harare, Zimbabwe: January 2017 to December 2018.

Characteristics	Started PEP	Did not complete PEP‡		RR (95% CI)	*p* value
	N	N	(%)		
**Total**	142	127	(89%)		
**Gender**					
Male	54	46	(85)	Ref	
Female	88	81	(92)	1.08 (0.95–1.23)	0.20
**Age group in years**					
< 20	3	3	(100)	1.04 (0.84–1.29)	0.83
20–29	72	67	(93)	Ref	
30–39	53	44	(83)	0.89 (0.78–1.02)	0.08
40–49	5	4	(80)	0.86 (0.55–1.34)	0.68
≥5 0	2	2	(100)	1.02 (0.75–1.39)	0.88
Missing data	7	7	(100)	-	
**Job title**					
Medical doctors	52	48	(92)	1.21 (0.96–1.54)	0.11
Medical students	13	13	(100)	1.31 (1.04–1.64)	0.15
Nurses	28	24	(86)	1.13 (0.86–1.47)	0.58
Student nurses / nurse aids	19	18	(95)	1.25 (0.98–1.59)	0.20
Dental / laboratory staff	5	5	(100)	1.29 (1.00–1.66)	0.65
Non clinical staff §	25	19	(76)	Ref	
**Type of occupational injury**					
Needle stick	105	94	(90)	0.97 (0.85–1.10)	0.63
Cuts (blades, glass)	8	6	(75)	0.81 (0.54–1.23)	0.44
Splashes	27	25	(93)	Ref	
Human bite	1	1	(100)	0.98 (0.54–1.79)	0.93
Missing data	1	1	(100)	-	

‡ Did not complete PEP = either did not take the full 28 days or there was missing data on PEP duration;

§: includes cleaners, porters, messengers and others;

PEP = post-exposure prophylaxis; RR = relative risk; CI = confidence interval.

## References

[1] Cardo DM, Culver DH, Ciesielski CA, Srivastava PU, Marcus R, Abiteboul D, Heptonstall J, Ippolito G, Lot F, McKibben PS, Bell DM (1997) A case-control study of HIV seroconversion in health care workers after percutaneous exposure. N Engl J Med 337: 1485–1490. 9366579 10.1056/NEJM199711203372101

[2] Landovitz RJ, Currier JS (2009) Postexposure prophylaxis for HIV infection. New Engl J Med 361: 1768–1775. 19864675 10.1056/NEJMcp0904189

[3] World Health Organization (WHO) (2014) Guidelines on post-exposure prophylaxis for HIV and the use of co-trimoxazole prophylaxis for HIV-related infections among adults, adolescents and children. Recommendations for a public health approach: 12 2014 supplement to the 2013 Consolidated ARV Guidelines. Geneva, Switzerland (ISBN 9789241506830). 26042326

[4] World Health Organization (WHO) (2016) Consolidated Guidelines on the use of antiretroviral drugs for treating and preventing HIV infection. Recommendations for a public health approach. Second Edition. Geneva, Switzerland (ISBN 9789241549684). 27466667

[5] World Health Organization (WHO) (2018) Updated recommendations on first-line and second-line antiretroviral regimens and post-exposure prophylaxis and recommendations on early infant diagnosis of HIV. Supplement to the 2016 consolidated guidelines on the use of antiretroviral drugs for treating and preventing HIV infection. Geneva, Switzerland (WHO/CDS/HIV/18.51).

[6] Siedner MJ, Tumarkin E, Bogoch II (2018) HIV post-exposure prophylaxis (PEP). BMJ 363: k4928. 30498074 10.1136/bmj.k4928

[7] Van Oosterhout JJG, Nyirenda M, Beadsworth MB, Kanyangalika JK, Kumwenda JJ, Zijlstra EE (2007) Challenges in HIV post-exposure prophylaxis for occupational injuries in a large teaching hospital in Malawi. Trop Doctor 37: 4–6. 10.1258/004947507779951961 17326876

[8] Mabwe P, Kessy AT, Semali I (2017) Understanding the magnitude of occupational exposure to human immunodeficiency virus (HIV) and uptake of HIV post-exposure prophylaxis among health care workers in a rural district in Tanzania. J Hosp Infect 96: 276–280. 28274607 10.1016/j.jhin.2015.04.024

[9] Rossouw TM, Van Rooyen M, Richter KL (2017) Exposure incidents among medical students in a high-prevalence HIV setting. J Infect Dev Ctries 11: 65–72. doi: 10.3855/jidc.8940. 28141592

[10] Bareki P, Tenego T (2018) Assessment of knowledge, attitudes and practices of HIV post exposure prophylaxis among the doctors and nurses in Princess Marina Hospital, Gaborone: a cross-sectional study. Pan Afr Med J 30: 233. 10.11604/pamj.2018.30.233.10556 PMC6295310 30574252

[11] Ajayi AI, Ismail KO, Adeniyi OV, Akpan W (2018) Awareness and use of pre-occupational injury and postexposure prophylaxes among Nigerian university students: findings from a cross-sectional survey. Medicine (Baltimore) 97: e12226. 30200145 10.1097/MD.0000000000012226 PMC6133481

[12] Kimaro L, Adinan J, Damian DJ, Njau B (2018) Prevalence of occupational injuries and knowledge of availability and utlilization of post exposure prophylaxis among health care workers in Singida District Council, Singida Region, Tanzania. PLOS ONE 13: e0201695. 30359370 10.1371/journal.pone.0201695 PMC6201876

[13] Aigbodion SJ, Motara F, Laher AE (2019) Occupational blood and body fluid exposures and human immunodeficiency virus post-exposure prophylaxis amongst intern doctors. S Afr J HIV Med 20: a958. 10.4102/HIVMED.v20i1.958 PMC6561641 31205779

[14] Tapesana S, Chirundu D, Shambira G, Gombe NT, Juru TP, Mufuta T (2017) Clinical care given to victims of sexual assault at Kadoma General Hospital, Zimbabwe: a secondary data analysis, 2016. BMC Infect Dis 17: 602. 28859613 10.1186/s12879-017-2702-4 PMC5580318

[15] Zimbabwe National Statistics Agency (ZIMSTAT) (2015). Population Projections Thematic Report Census 2012. Harare: ZIMSTAT UNFPA 134p.

[16] The World Bank (2016). Gross Domestic Product (GDP) per capita (current US$). Available: http://data.worldbank.org/indicator/NY.GDP.PCAP.CD?view=chart. Accessed: 10 November 2019.

[17] Columbia University (2015–2016) Zimbabwe Population-based HIV Impact Assessment (ZIMPHIA). Available: https://phia.icap.columbia.edu/wp-content/uploads/2016/11/ZIMBABWE-Factsheet.FIN_.pdf. Accessed: 10 November 2019.

[18] Ministry of Health and Child Care (2013). National Infection Prevention and Control Guidelines. Harare: Ministry of Health and Child Care 157p.

[19] Ministry of Health and Child Care (2016) Guidelines for Antiretroviral therapy for the Prevention and Treatment of HIV in Zimbabwe. Harare: National Medicines and Therapeutics Policy Advisory Committee (NMTPAC) and the AIDS and TB Directorate, Ministry of Health and Child Care (MOHCC) 136p.

[20] Abubakar S, Iliyasu G, Dayyab FM, Inuwa S, Tudun Wada RA, Sadiq NM, Gadanya MA, Sheshe AA, Mijinyawa MS, Haib AG (2018) Post-exposure prophylaxis following occupational injury to HIV and hepatitis B: an analysis of a 12-year record in a Nigerian tertiary hospital. J Infect Prev 19: 184–189. 30013623 10.1177/1757177417746733 PMC6039908

[21] Braczkowska B, Kowalska M, Beniowski M, Zejda JE, Mazur W, Witor A (2010) Occupational exposure to HIV in health care workers, Silesia voivodeship. Med Pr 61: 315–322. [Article in Polish] 20677431

[22] Domkam IK, Sonela N, Kamgaing N, Takam PS, Gwom LC, Betilene TMA, Fokam J, Billong SC, Moukam LV, Etounou TM, Minka CSM, Ndjolo A (2018) Prevalence and risk factors to HIV infection amongst health care workers within public and private health facilities in Cameroon. Pan Afr Med J 29: 158. 30050622 10.11604/pamj.2018.29.158.14073 PMC6057590

[23] Ajibola S, Akinbami A, Elikwu C, Odesanya M, Uche E (2014) Knowledge, attitudes and practices of HIV post exposure prophylaxis amongst health care workers in Lagos University Teaching Hospital. Pan Afr Med J 19:172. 25815093 10.11604/pamj.2014.19.172.4718 PMC4366120

[24] Kassa G, Selenic D, Lahuerta M, Gaolathe T, Liu Y, Letang G, Courtney-Quirk C, Mwanki NK, Gaolekwe S, Bock N (2016) Occupational exposure to bloodborne pathogens among health care workers in Botswana: reporting and utilization of postexposure prophylaxis. Am J Infect Control 44: 879–885. 27021510 10.1016/j.ajic.2016.01.027

[25] Papavarnavas NP, Manning K, Conrad F, Govender M, Maartens G (2017) Factors associated with loss to follow-up after occupational HIV exposure in Cape Town, South Africa: a retrospective cohort study. AIDS Res Ther 14: 23. 28431556 10.1186/s12981-017-0149-8 PMC5401471

[26] von Elm E, Altman DG, Egger M, Pocock SJ, Gotzsche PC, Vandenbroucke JP; STROBE initiative (2007) The Strengthening the Reporting of Observational Studies in Epidemiology (STROBE) statement: guidelines for reporting observational studies. Bull World Health Organ 85: 867–72. 18038077 10.2471/BLT.07.045120 PMC2636253

[27] Kannisto KA, Koivunen MH, Valimaki MA (2014) Use of mobile phone text message reminders in health care services: a narrative literature review. J Med Internet Res 16: e222. 25326646 10.2196/jmir.3442 PMC4211035

[28] Bertman V, Petracca F, Makunike-Chikwinya B, Jonga A, Dupwa B, Jenami N, Nartker A, Wall L, Reason L, Kundhlande P, Downer A (2019) Health worker text messaging for blended learning, peer support, and mentoring in pediatric and adolescent HIV/AIDS care: a case study in Zimbabwe. Hum Resour Health 17: 41. 31174543 10.1186/s12960-019-0364-6 PMC6555929

[29] Indravudh PP, Choko AT, Corbett EL (2018) Scaling up HIV self-testing in sub-Saharan Africa: a review of technology, policy and evidence. Curr Opin Infect Dis 31: 14–24. 29232277 10.1097/QCO.0000000000000426 PMC5768229

[30] Courtenay-Quirk C, Selenic D, Lahuerta M, Kassa G, Murrman M, Bock N (2016) Development of an intervention to increase occupational postexposure prophylaxis in sub-Saharan Africa. J Assoc Nurses AIDS Care 27: 727–730. 27425796 10.1016/j.jana.2016.06.004 PMC4981482

